# Predictors of Birth Preparedness and Complication Readiness Practices Among Pregnant Women in Ethiopia, a Systematic Review and Meta-Analysis

**DOI:** 10.3389/ijph.2024.1607296

**Published:** 2024-09-02

**Authors:** Abebaw Alamrew, Mulat Ayele, Eyob Shitie Lake, Chalie Mulugeta, Getinet Kumie, Alemu Birara Zemariam

**Affiliations:** ^1^ Department of Midwifery, College of Health Sciences, Woldia University, Woldia, Ethiopia; ^2^ Department of Medical Laboratory Science, College of Health Sciences, Woldia University, Woldia, Ethiopia; ^3^ Department of Pediatrics and Child Health Nursing, School of Nursings, College of Medicine and Health Science, Woldia University, Woldia, Ethiopia

**Keywords:** birth preparedness, complication readiness, pregnancy, knowledge of danger signs, Ethiopia

## Abstract

**Objectives:**

We conducted this review to identify factors associated with birth preparedness and complication readiness (BPCR) among pregnant women in Ethiopia. BPCR is a comprehensive approach that helps address delays in seeking care for obstetric problems.

**Methods:**

PRISMA was followed and different databases were used to find studies. Adjusted Odds Ratio (AOR) with a 95% Confidence Interval was used to identify factors. The I^2^ statistic, funnel plot, and Egger test were used to assess the heterogeneity of studies and publication bias.

**Results:**

Knowledge of BPCR, danger signs during pregnancy, labor, and postpartum (AOR = 1.99, 95% CI: 1.51, 2.64, AOR = 1.55; 95% CI: 1.35, 1.80; AOR = 1.45; 95% CI: 1.27, 1.63, and AOR = 1.4; 95% CI: 1.21, 1.63), respectively, residency (AOR = 1.49; 95% CI: 1.32, 1.68), antenatal care visit (AOR = 1.59; 95% CI: 1.43, 1.78), history of stillbirth (AOR = 1.58; 95% CI: 1.36, 1.86), and educational status (AOR = 1.62: 95% CI: 1.45, 1.78) were significantly associated with BPCR practice.

**Conclusion:**

This study identified some modifiable factors in the practice of BPCR. Integrating counseling and expanding ANC services in health facilities may improve BPCR practice.

## Introduction

Pregnant women are vulnerable to problems that emerge during pregnancy and childbirth and can end in death at any time, anywhere in the world. Worldwide, 800 women die every day; 95% of these deaths take place in low- and middle-income nations; Sub-Saharan Africa and Southern Asia account for approximately 87% of the expected total number of global maternal deaths, and Sub-Saharan Africa alone accounts for approximately 70% of these deaths [1, 2]. By implementing comprehensive maternal health services, the majority of these deaths can be avoided [3].

Pregnancy, childbirth, postpartum period, and neonatal care are all included in birth preparedness and complication readiness, or BPCR, which is the state of being ready for delivery and any obstetric emergency [4, 5]. The Johns Hopkins Program for International Education in Gynecology and Obstetrics (JHIEGO) maternal and neonatal health program describes BPCR as focusing on three delays: delays in making the decision to seek care for an obstetric complication, delays in going to a medical facility after making the decision, and delays in receiving care at the facility [6]. Its components include identifying a place to give birth, identifying a skilled birth attendant, saving money for birth-related and emergency expenses, identifying a support person during pregnancy, labor, and postpartum periods, identifying means of transport in case of an obstetric emergency, preparing equipment needed for delivery and identifying a potential blood donor in case of an emergency [7, 8].

The UN Sustainable Development Goals (SDGs) of reducing maternal mortality to less than 70 per 100,000 live births, neonatal mortality to at least 12 per 1,000 live births, and under-five mortality to at least as low as 25 per 1,000 live births can be greatly supported and achieved by implementing BPCR, especially in developed nations [7, 9, 10]. Ethiopia as a member country has adopted these goals and has been giving great attention and efforts to achieve the target by 2030. In Ethiopia maternal, neonatal, and under-five mortality rates have decreased, although they are still high and far from the target of the SDGs [10]. Still, 1 in 267 women dies from pregnancy-related complications, 1 in 20 children dies before reaching the age of 5, 1 in 28 infants dies before reaching 1 year of age, and 1 in 38 newborns dies within their first 4 weeks of life which is still too high [11, 12].

Research findings revealed that BPCR therapies contributed to a decreased incidence of adverse outcomes for both mothers and neonates. Specifically, women who practice BPCR had lower rates of low birth weight neonates (4.3% vs. 14.8%), admission to a neonatal intensive care unit (14.8% vs. 32.2%), and postpartum anemia (35.7% vs. 58.3%) [4, 13, 14]. Other study results indicated that BPCR interventions contributed to a 28% reduction in the risk of maternal mortality and an 18% reduction in the risk of neonatal mortality [4].

Birth preparedness and complication readiness is a comprehensive method for increasing the timely utilization of skilled maternal and neonatal care [2, 7]. Although there is clear evidence that birth preparedness and complication readiness can improve maternal and neonatal health outcomes, national data on factors associated with BPCR practices BPCR are scarce, and regional variations exist [9, 15–36]. In Ethiopia, one study has been conducted at the national level on the practice of birth preparedness and complication readiness plans [37], but comprehensive studies on the predictors of BPCR practices at the national level are lacking, except for small-scale studies. Therefore, this systematic review and meta-analysis aimed to identify predictors of birth preparedness and complication readiness practices among pregnant mothers in Ethiopia.

## Methods

### Development of the Review Methodology

The Preferred Reporting Elements for Systematic Reviews and Meta-Analyses Protocols (PRISMA-P) 2020 statement was used to develop the methodology of the review, and all items of the PRISMA-P checklist were covered (Supplementary Figure 1). The PRISMA flow charts showing the study selection process from the first identified records to the included studies, were noted in the results. The International Prospective Register of Systematic Reviews (PROSPERO) delivered registration number CRD42023442256 for this systematic review and meta-analysis.

### Search Date

The initial searches for this systematic review were started on 01/07/2023, and the protocol was registered on 15/07/2023. This systematic review and meta-analysis includes all studies published in Ethiopia until 24/10/2023.

### Search Strategy

The first author (AA) conducted the literature search. Only research that was published in English was included in the search. We used various combinations of keywords to search in the databases and to find articles for the review, major medical electronic databases including MEDLINE, PubMed, Google Scholar, CINAHL, EMBASE, Scopus, and African Journals Online were used. The search strings or terms stemmed from the following keywords: birth preparedness, complication readiness, knowledge of danger signs, associated factors, and Ethiopia. A sample of the PubMed search strategy (“factors$ or determinants) and (“birth preparedness”) and (complication readiness$ or “knowledge of danger signs”) (pregnant$ or “prenatal”).

### Eligibility Criteria

We considered studies that examined the prevalence and associated factors of BPCR. Regardless of the time of data collection or year of publication, we included studies that had been conducted in both community and facility settings, with the primary outcome variable being characterized as “practice of BPCR and its associated factors among pregnant women” in the analysis. Case series, case reports, reviews, and studies that did not record the outcome variable were not included. Research that primarily provided qualitative insights into the use of BPCR was also excluded. Only quantitative data from studies that reported both qualitative and quantitative findings were taken into consideration.

### Study Selection Procedure

#### Screening

**FIGURE 1:**
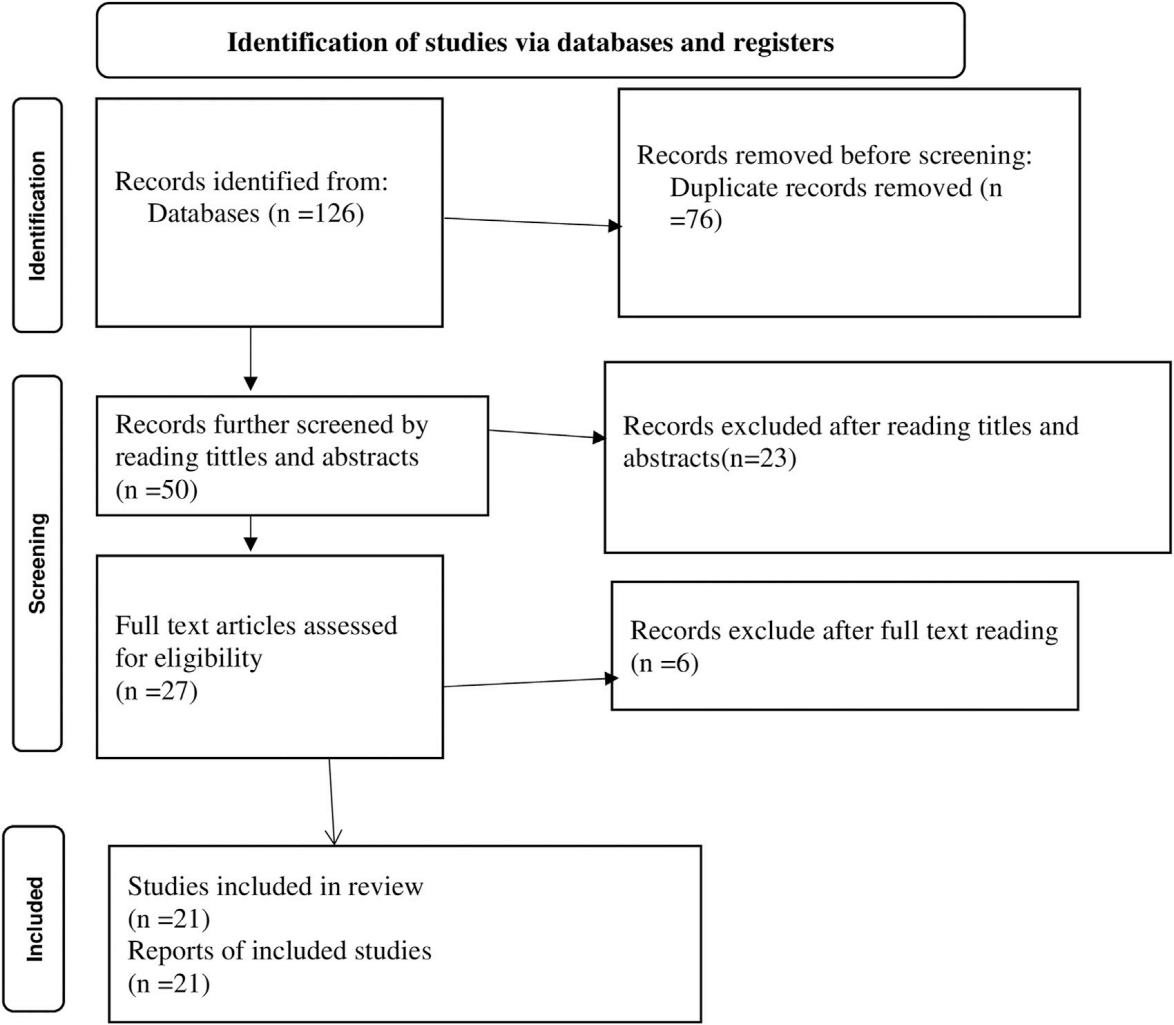
PRISMA flow chart showing the included searches for systematic review and meta-analysis on predictors of birth preparedness and complication readiness practice among pregnant women in Ethiopia, 2023.

Initial studies were identified using search terms and filters in databases and other relevant sources. Duplicate studies were removed from the identified studies after exporting them to EndNote, a citation management tool. According to the inclusion criteria, the studies were independently screened by four authors (MA, CM, GK, and ES) using the data from the titles and abstracts. The titles and abstracts of the studies were categorized as included, excluded, or undetermined based on this screening process. For a more thorough eligibility evaluation, we then obtained the full text of each included and inconclusive study (Figure 1).

#### Quality Assessment

Two authors (AA and MA) evaluated the methodological quality, risk of bias, and validity of the included articles by using the Joanna Briggs Institute (JBI) checklist designed for analytical cross-sectional studies. There are eight questions on the checklist (1. Are the criteria for inclusion in the sample clearly defined? 2. Were the study subjects and the setting described in detail? 3. Was the exposure measured validly and reliably? 4. Were objective, standardized criteria used for the measurement of the condition? 5. Were confounding factors identified? 6. Were strategies for dealing with confounding factors stated? 7. Were outcomes measured validly and reliably? and 8. Was an appropriate statistical analysis used?), categorized into “Yes,” “No,” “Unclear” and “Not applicable.” Scores of 50% and above on the quality assessment indicators were considered low risk [38]. Any disagreement over the assessment of the risk of bias and research quality between the two authors was managed by involving the third and fourth authors (ES and GK).

#### Outcome Measurements

The out comes of this review was identification of factors affecting birth preparedness and complication readiness practices among pregnant women in Ethiopia which were measured by identifying the place of delivery, saving money for delivery, identifying a mode of transportation to the place of delivery or in case of emergency, identifying a potential blood donor, identifying a skilled birth provider, and identifying a support person or decision maker. Factors that could be involved in BPCR practices were measured as higher levels of maternal educational status (yes/no), maternal residence (urban/rural), government employee (yes/no), knowledge of BPCR (yes/no), knowing danger signs during pregnancy (yes/no), knowing danger signs during labor and delivery (yes/no, knowing danger signs during the post-natal period (yes/no), having antenatal care follow-up during pregnancy (yes/no) and history of stillbirth (yes/no).

#### Data Extraction Process

The data were meticulously abstracted by two authors (AA and CM) using a data extraction template. To represent all the included research, a list of items relevant to the study characteristics is presented in the summary table. Names of the authors, study year, design, setting, sample size, demographics, and study-specific predictive factors were all included in the detailed list of items. Throughout the data extraction procedure for the exposure variables, we separated the individual classifications displayed for each variable into two categories: exposed and non-exposed to the outcome. For the variables, the non-exposed group served as the reference. For example, the exposed category for place of residence was rural, whereas the non-exposed group was urban. We then prepared them for quantitative synthesis using the matching combined numerical values. After face-to-face discussions, disagreements between the two authors were resolved and an agreement was reached.

#### Data Synthesis and Statistical Analysis

A summary table was used to present the main findings of the included articles. Additionally, Stata 17 was used for analysis. We used 95% confidence intervals (CIs) for adjusted odds ratios (AORs) to compute summary statistics (pooled effect sizes). The results of the meta-analysis were graphically shown using forest plots. The I^2^ statistic was used to evaluate statistical heterogeneity and the degree of heterogeneity between the studies; if a value was ≥50%, significant heterogeneity was considered and we performed sub-group analysis based on study region, study design, and year of publication. Egger’s test was used to test the publication bias by considering a *P*-value: a *P*-value of <0.05 meant that there was potential publication bias or small-study effects, and a *P*-value ≥ 0.05 suggested the absence of strong publication bias or small-study effects [39].

## Results

### Description of the Studies

A search of primary electronic health and medical databases along with other relevant sources identified 126 studies. Based on their title and abstract, 50 of the identified studies were kept for further eligibility screening, while the remaining 76 were excluded as duplicates. After reviewing the titles and abstracts of 23 records, it was determined that 9 studies did not meet the inclusion criteria and 14 studies were conducted in a location or environment unrelated to the research question. Six of the 27 papers that remained were eliminated after their full texts were reviewed because, as indicated by their titles, abstracts, and texts, their content did not relate to the topic of our evaluation. After thorough evaluation, the remaining 21 studies were included in this review (Figure 1). Seven institutional-based and fourteen community-based cross-sectional studies with a total sample size of 13,877 were included in this review. Of the included studies nine were from the SNNP region, four were from the Amhara region, five were from the Oromia region, two were from the Harare region, and one was from the Afar region (Table 1).

**TABLE 1:** Description of the included studies to identify predictors of birth preparedness and complication readiness practice among pregnant women in Ethiopia, 2023.

Author’s name, year	Study region	Study design	Sample size	Study participants	Factors investigated in the study
Endeshaw et al., 2018 [36]	Amhara	Community-based cross-sectional	507	500	Place of residence, Knowledge of BPCR, danger signs during pregnancy, initiation of ANC, Gestational age
Limenih et al., 2019 [40]	Amhara	Community-based cross-sectional	676	676	Place of residence, educational status, Knowledge of danger signs during pregnancy, labor and delivery, postpartum, Knowledge of BPCR, antenatal care follow-up, and history of stillbirth
Bitew et al., 2016 [3]	Amhara	Community-based cross-sectional	845	819	Educational status, Knowledge of BPCR, history of still birth, trimester of pregnancy, Male partner involvement and ANC follow history of Stillbirth, Knowledge of danger signs during pregnancy, labor, and delivery
Asefa et al., 2019 [26]	Amhara	Institution-based cross-sectional	340	340	Knowledge of danger signs during pregnancy, labor, and delivery and stillbirth
Bejitual and Jabessa, 2019 [24]	Oromia	Institution-based cross-sectional	273	272	Educational status, occupational status, Knowledge of danger signs during pregnancy, labor, and delivery, and knowledge of BPCR in women aged 20–24 and 25–29
Gurmesa Tura Debelew et al,2012 [9]	Oromia	Community-based cross-sectional	3,612	3,612	Place of residence, Educational status, occupational status, ANC follow-up, Knowledge of danger signs during labor and delivery, Attitude towards BPCR
Gedefa et al., 2020 [33]	Oromia	Community-based cross-sectional	310	307	Educational status, occupational status, ANC follow-up, gravidity, Knowledge of danger signs during pregnancy, Knowledge of danger signs during labor and delivery, Knowledge of danger signs during postpartum, and Knowledge of BPCR
Kaso and Addisse, 2012 [21]	Oromia	Community-based cross-sectional	581	575	Monthly income, educational status, Knowledge of danger signs during pregnancy, Knowledge of danger signs during labor and delivery, Knowledge of danger signs during postpartum, ANC follow up, Birth at a health facility before last delivery, birth order, parity, and gravidity
Markos and Bogale, 2014 [41]	Oromia	Community-based cross-sectional	580	562	Place of residence, Educational status, Knowledge of danger signs during pregnancy, after delivery, and ANC follow-up
Begashaw et al., 2017 [30]	SNNP	Institution-based cross-sectional	397	392	Income, History of Stillbirth
Gebre et al., 2015 [15]	SNNP	Community-based cross-sectional	578	569	Knowledge of danger signs during pregnancy, postpartum ANC follow-up, History of stillbirth, parity
Halil et al., 2019 [2]	SNNP	Institution-based cross-sectional	250	250	Knowledge of danger signs during labor and delivery, Number of ANC visits during previous pregnancy
Mathewos et al., 2022 [27]	SNNP	Community-based cross-sectional	462	459	ANC follow-up and Knowledge of danger signs during labor and delivery
Gudeta and Regassa, 2019 [23]	SNNP	Community-based cross-sectional	605	605	Religion, income, place of residence, occupational status Knowledge of BPCR, Knowledge of danger signs during pregnancy in women aged 18–19 and 20–34
Gesese and Tirfe, 2020 [6]	SNNP	Institution-based cross-sectional	422	422	Educational status, ANC follow-up, belief that ANC is useful, history of delivery at a health facility in women aged 25–30 and 37–44
Wudu and Tsegaye, 2021 [32]	SNNP	Community-based cross-sectional	491	491	IEducational status, ANC follow-up, belief that ANC is useful, history of delivery at a health facility, women aged 25–30, and 37–44
Hailu et al., 2007 [18]	SNNP	Community-based cross-sectional	812	743	ANC follow-up and gravidity
Iyasu et al., 2018 [35]	SNNP	Community-based cross-sectional	746	746	Age, educational status of the husband, Knowledge of danger signs during pregnancy, labor, delivery, and after delivery
Ibrahim et al., 2020 [28]	Harare	Institution-based cross-sectional	419	403	Place of Residence, Knowledge of BPCR, Knowledge of danger signs during pregnancy, labor and delivery, planned pregnancy in women aged 20–34, or with age greater or equal to 35
Regesu et al., 2019 [25]	Harare	Institution-based cross-sectional	303	303	Occupational status, Knowledge of danger signs during pregnancy, labor, delivery and after delivery, ANC follow-up, interval between pregnancies
Ananche and Wodajo, 2020 [17]	Afar	Community-based cross-sectional	391	361	Religion, income, and Knowledge of BPCR in women aged 18–19 and 20–34

ANC, antenatal care visit; BPCR, Birth preparedness and complication readiness; SNNP, Southern Nation, Nationalities and Peoples’ Region.

### Factors Associated With BPCR Practices

The current review reveals various factors associated with BPCR practices in Ethiopia. Significantly associated factors include maternal knowledge of BPCR, maternal knowledge of pregnancy danger signs, maternal knowledge of labor and delivery danger signs, maternal knowledge of postpartum danger signs, place of residence, ANC follow-up, history of stillbirth, and maternal education.

### Place of Residence

**FIGURE 2:**
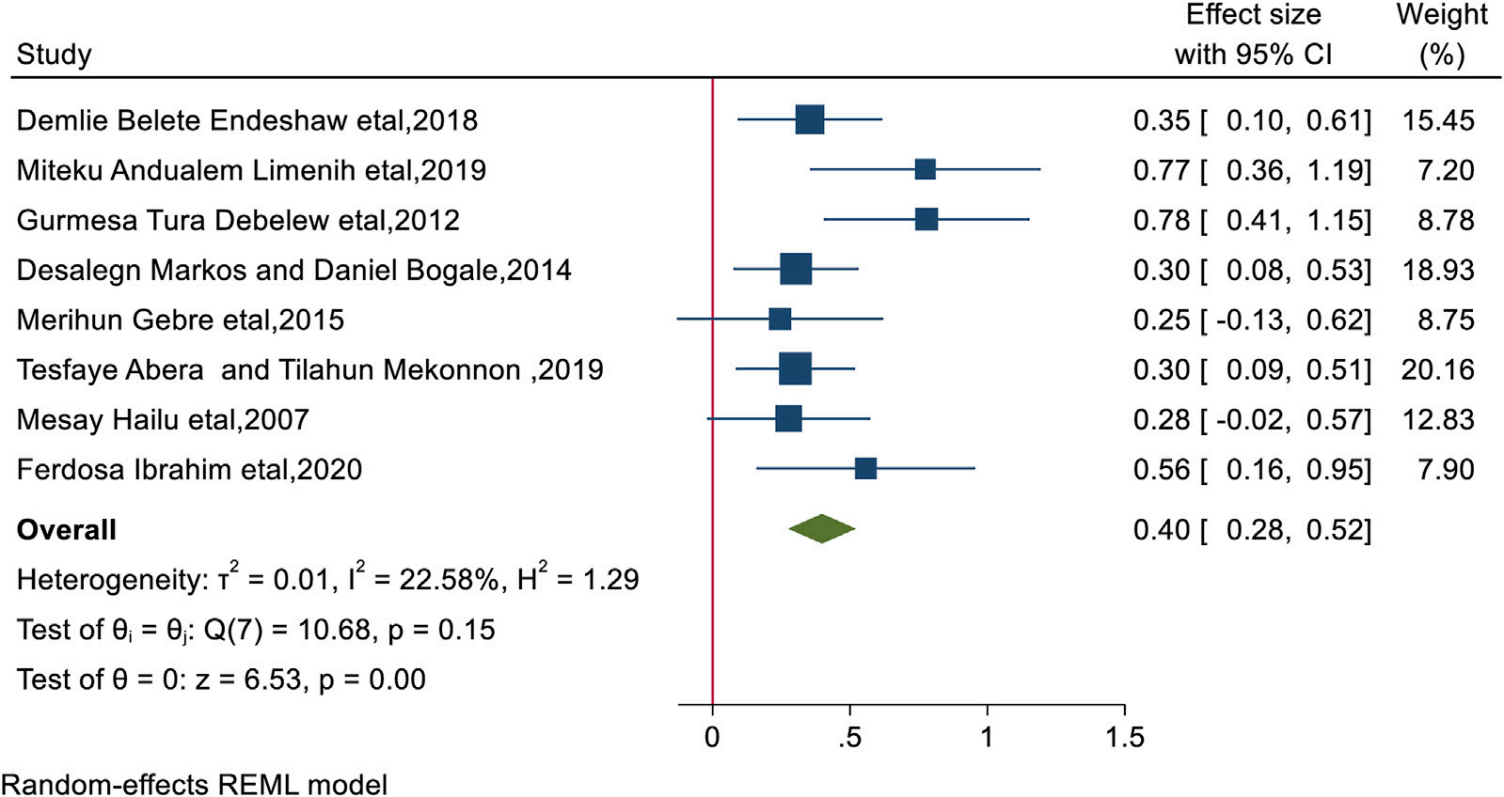
The overall pooled odds ratio of the association between residency and practice of birth preparedness and complication among pregnant women in Ethiopia, 2023.

Factor analysis of the included studies revealed a significant positive association between BPCR practices and place of residence. Women who lived in urban areas had a 1.49-fold higher chance of practicing BPCR than their counterparts (AOR = 1.49; 95% CI: 1.32, 1.68). This included the heterogeneity of the study (I^2^ = 22.58%, *p* < 0.0001) (Figure 2), and the Egger test revealed publication bias with a *p*-value of 0.0223. For this, we conducted a nonparametric trim-and-fill analysis of publication bias and this showed no imputed study and we report with the original finding.

### Maternal Education

**FIGURE 3:**
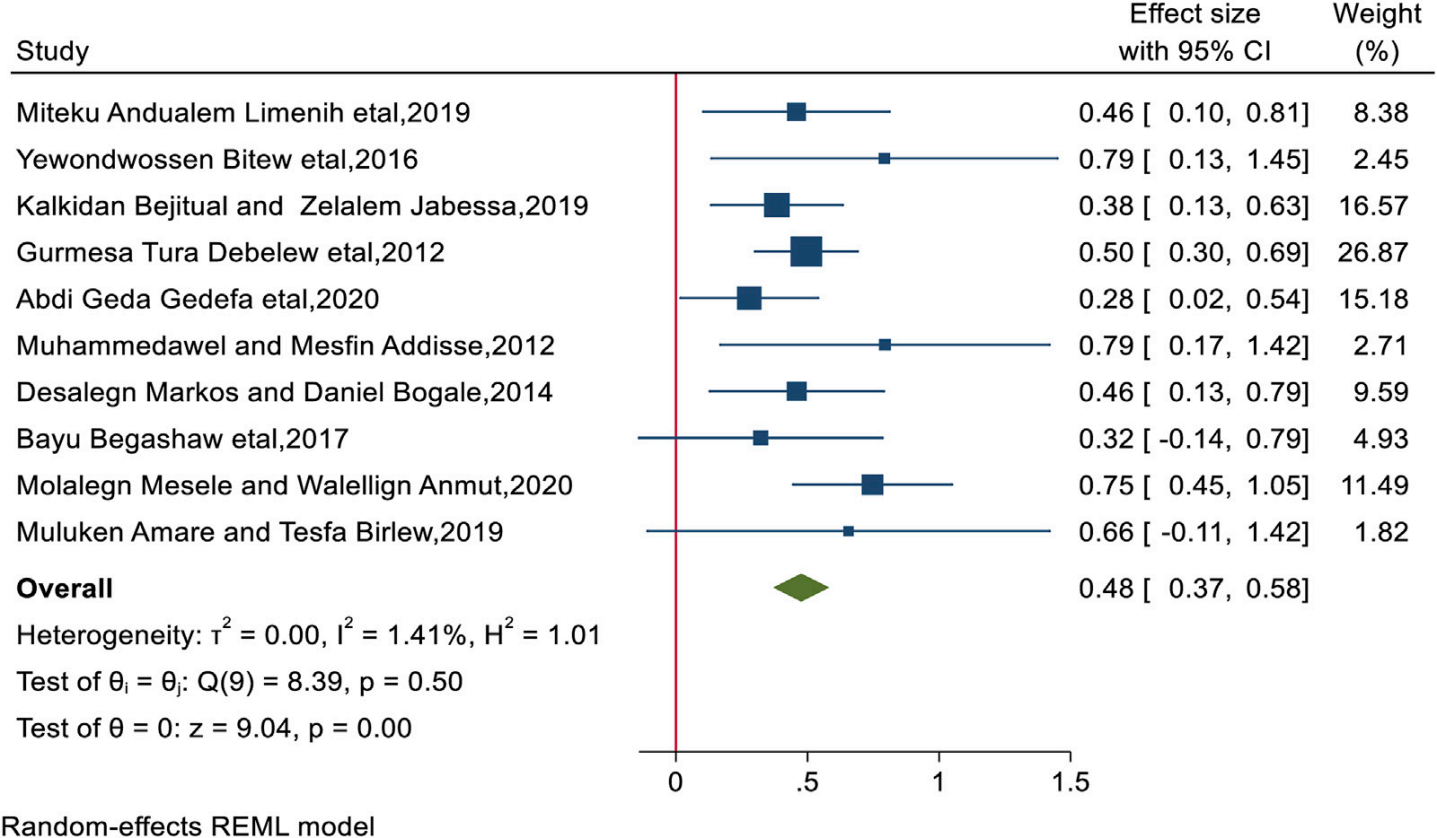
The overall pooled odds ratio of the association between the mother’s educational status and practice of birth preparedness and complication readiness among pregnant women in Ethiopia, 2023.

According to this study, maternal education was found to be associated positively with BPCR use, with women who completed secondary education or higher adopting BPCR practices 1.62 times more than their counterparts (AOR = 1.62: 95% CI: 1.45, 1.78). Significant heterogeneity was not detected in included study (I^2^ = 1.41%, *p* < 0.0001), and the Egger test revealed no publication bias with a *p*-value of 0.27 (Figure 3).

### Maternal Knowledge of BPCR

**FIGURE 4:**
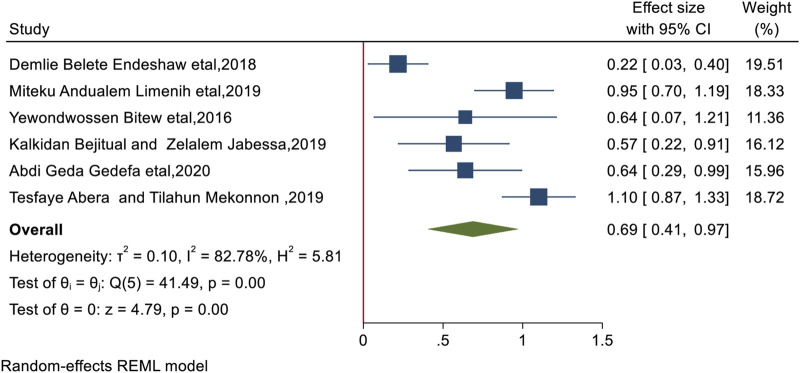
The overall pooled odds ratio of the association between knowledge of birth preparedness and complication readiness and practice of birth preparedness and complication readiness among pregnant women in Ethiopia, 2023.

BPCR practices were found to be significantly positively correlated with maternal knowledge of BPCR. This review showed that women who knew about BPCR were nearly twice as likely to practice BPCR as women who did not (AOR = 1.99, 95% CI: 1.51, 2.64). There was significant heterogeneity (I^2^ = 82.78%, *p* < 0.0001). The Egger test yields a *p*-value of 0.969, indicating no publication bias (Figure 4).

### Antenatal Care Visit

**FIGURE 5:**
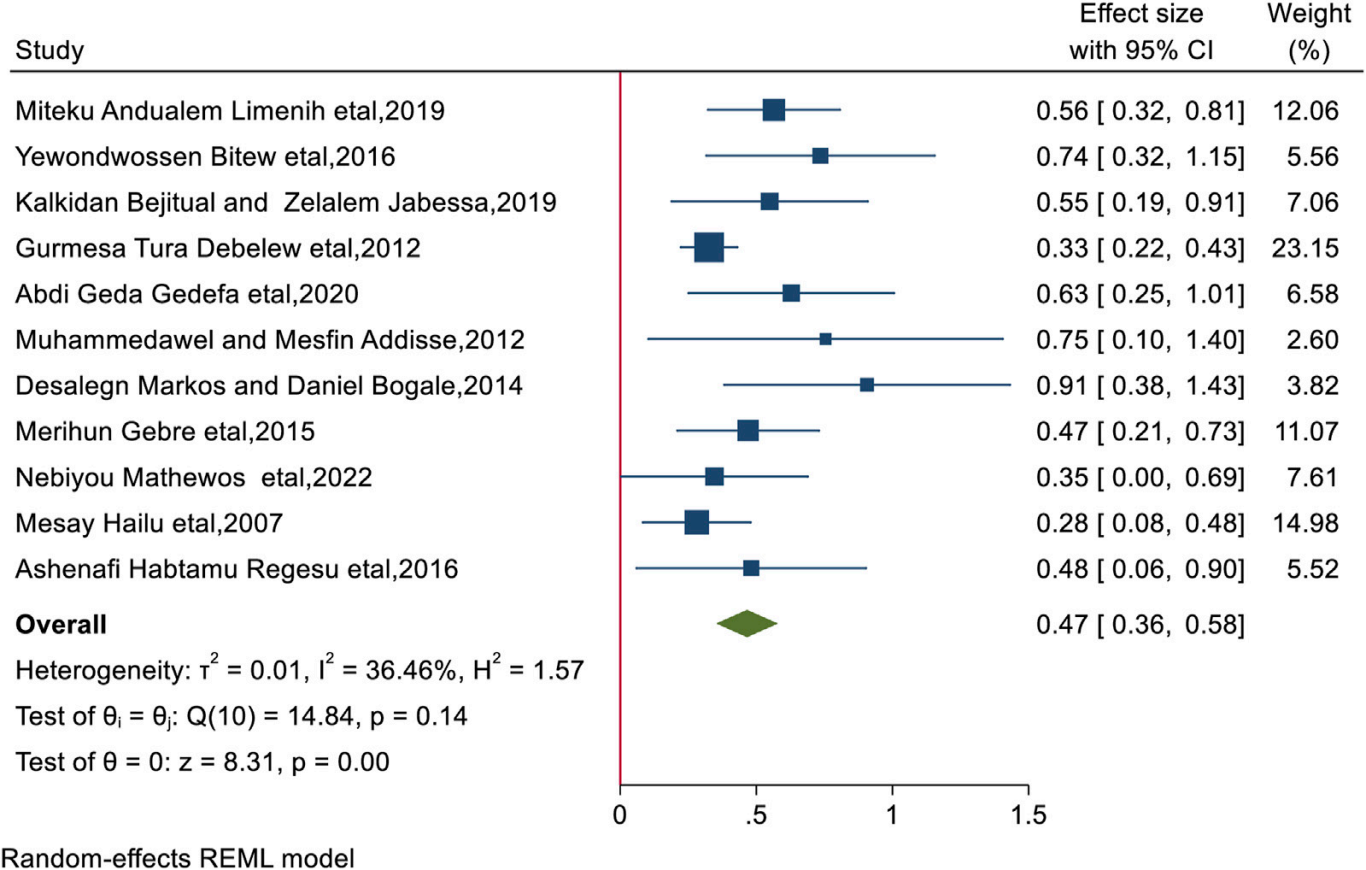
The overall pooled odds ratio of the association between antenatal care follow-up and practice of birth preparedness and complication readiness among pregnant women in Ethiopia, 2023.

The conclusions of the review showed that women who received ANC visits had a much higher likelihood of practicing BPCR than their counterparts (AOR = 1.59; 95% CI: 1.43, 1.78), women who received ANC visits were 1.59 times more likely to practice BPCR than their counterparts. The included studies had no significant heterogeneity (I^2^ = 36.46%, *p* < 0.0001), while the Egger test revealed publication bias with a significant *p*-value of 0.0018, for which we performed a nonparametric trim-and-fill analysis of publication bias, indicating no imputed study (Figure 5).

### History of Stillbirth

According to this review, women who had previously experienced a stillbirth had a higher likelihood of using BPCR than other women (AOR = 1.58; 95% CI: 1.36, 1.86). Women having a history of stillbirths were 1.58 times more likely to practice BPCR. The heterogeneity of this included study was low (I^2^ = 8.35%, *p* < 0.0001) and there is no publication bias using the Egger test with a nonsignificant *p*-value of 0.37 (Supplementary Figure 2).

### Knowledge of Danger Signs During Pregnancy

The overall odds ratio (AOR = 1.55; 95% CI: 1.35, 1.80) indicated that women who knew the danger signs during pregnancy were 1.55 times more likely to practice BPCR than women who did not know the signs during pregnancy. The heterogeneity of the study was high (I^2^ = 82.78%, *p* < 0.0001) and the Egger test yielded a *p*-value of 0.46 indicating no publication bias (Supplementary Figure 3).

### Knowledge of Danger Signs During Labor and Delivery

The findings of the review showed a significant positive association between women’s knowledge of danger signs during labor and delivery and their likelihood of practicing BPCR (AOR = 1.45; 95% CI: 1.27, 1.63), with women who were aware of the warning signs during labor and delivery being 1.45 times more likely to use BPCR than those who were not. The Egger test yielded a *p*-value of 0.54, with no indication of publication bias, and heterogeneity was found in the included studies (I^2^ = 69.26%, *p* < 0.0001) (Supplementary Figure 4).

### Knowledge of Postpartum Danger Signs

Our analysis revealed a significant positive association between women’s knowledge of postpartum danger signs and BPCR practice (AOR = 1.4; 95% CI: 1.21, 1.63), with women who were aware of postpartum danger signs being 1.4 times more likely to practice BPCR than women who were not aware. This accounted for the heterogeneity of the study (I^2^ = 76.87%, *p* < 0.0001), and the Egger test revealed no publication bias with a *p*-value of 0.46 (Supplementary Figure 5).

### Parity, Partner Involvement

Our systematic review showed no significant association between parity, partner involvement, and BPCR practice (OR = 0.59; 95% CI: −0.16, 1.34) and (OR = 0.65; 95% CI: −0.18, 1.47), respectively.

### Subgroup Analysis

When the I^2^ was greater than 50% it indicated high heterogeneity, we performed a subgroup analysis to identify the sources of heterogeneity (differences) in the study results. In this review, we performed a subgroup analysis by considering the study region (Amhara, SNNP, Oromia, Harare) and study design (community-based vs. institutional). Following the sub-group analysis, the study with the highest I^2^ showed the source of heterogeneity (Supplementary Figure 6).

### Publication Bias

In this systematic review and meta-analysis, we assessed the publication bias by performing the Egger test and funnel plot (Supplementary Figure 7) and we considered no publication bias to exist if the value of the Egger test was non-significant or if the value of *P* was ≥0.05. In this review of factor analysis, the value of the Eggers test was found to be significant and the funnel plot was asymmetrically distributed on the factors of place of residence and ANC visit. For this we conducted a trim-and-fill analysis, which indicated zero imputed studies. Consequently, we report only the observed findings for the final results.

## Discussion

This systematic review and meta-analysis aimed to identify the predictors of birth preparedness and complication readiness plans among Ethiopian women. Twenty-one studies were included in this meta-analysis. Maternal knowledge of BPCR plans, danger signs during pregnancy, labor, and postpartum, maternal education, maternal, place of residence, history of stillbirth, and ANC visits were significantly associated with BPCR practice in Ethiopia. Parity and paternal involvement in the BPCR showed no significant association with BPCR practices.

This systematic review and meta-analysis revealed that educational status and residence were significantly associated with BPCR practice. Urban women were more likely to practice BPCR than rural women; this is supported by a study conducted in Northern Ghana [42]. Urban women’s higher media exposure, practical access to healthcare facilities, and strong understanding of health issues may explain this. Women who have secondary and above education were also more likely to practice BPCR which is supported by other studies conducted in different countries [5, 42–52]. Women who have received education are more likely to seek medical attention, and it also helps them become confident and effective decision-makers, improving their readiness for childbirth and complications. Living in urban areas and receiving an education broadens awareness of obstetric difficulties and gives one access to BP/CR health information, allowing them to practice being ready for birth and for complications. Despite the above evidence, a study conducted in rural areas of Indian mothers revealed that the educational status of the mothers is not associated with BPCR practices [53]. This might be explained by a difference in sample size and variation in the study area.

The findings of this systematic review and meta-analysis revealed that women who knew about birth preparedness and complication readiness plans were significantly associated with BPCR practices, which is supported by a study conducted in Nepal, and India [43, 54]. This may be explained by the fact that women are more likely to engage in active planning and take preventative actions to ensure the health of both the mother and the newborn when they know what to expect during pregnancy and how to prepare for any issues and may practice BPCR.

This systematic review and meta-analysis showed that women who had antenatal care follow-up and a history of stillbirth during pregnancy were other predictors of birth preparedness and complication readiness practice, which is supported by studies conducted in Nepal, Kenya, Tanzania, and the Pakistani province of Sindh [43, 46, 55]. This may be explained by the fact that antenatal care visits provide mothers with essential knowledge regarding the significance of getting ready for childbirth, recognizing warning signs of problems, and knowing what to do in an emergency during these visits, and healthcare providers may look at the mother’s and the baby’s health and address any issues.

Antenatal care follow-up minimizes the risks associated with birthing and increases the use of BPCR by empowering pregnant moms by offering more knowledge and preparation. A history of stillbirth has been associated with the practice of BPCR. This could be explained by the fact that women who have had stillbirths tend to be more aware and optimistic during their subsequent pregnancies. This sensitive awareness usually motivates more careful participation in behaviors related to complication readiness and birth preparedness. They might be more likely to adhere to antenatal care plans, carefully examine and comply with medical advice, and take necessary precautions to avert any difficulties. As a result, a history of stillbirth can serve as a predictor for better antenatal care in the future, preventing recurrence.

This systematic review and meta-analysis showed that the odds of practicing BPCR were higher among women with knowledge of the danger signs during pregnancy. This is supported by studies conducted in Kenya and Tanzania [46], in the Kassena-Nankana district of Ghana [44], in resource-limited settings in rural northern Ghana [45], India [56], in the Thatta district, Sindh, Pakistan [55], in the urban slums of Shivamogga City, India [47], Nepal [43], and rural areas in Bangladesh [57] which could be explained by the fact that being aware of the danger signs associated with pregnancy can help women feel more prepared for giving birth, improve their behavior when seeking medical attention, and make better decisions regarding their health. It can also help identify potentially life-threatening complications early on and reduce unnecessary delays in seeking medical attention. This systematic review and meta-analysis also showed that the odds of practicing BPCR were higher among women who knew danger signs during labor and after giving birth. This finding is supported by studies conducted in Nepal, Kenya, Tanzania, Sindh, India, and Bangladesh [43, 46, 55–57]. It could be explained by the fact that women who are knowledgeable about childbirth, postpartum difficulties, labor, and delivery would be more worried about their safety and survival. Furthermore, women may be ready to avert the worst before it happens if they have greater awareness about delivery and postpartum risk signs. It also aids in their efforts to safeguard their safety and wellbeing, as well as the health of their child. This ultimately results in the need for medical guidance and support from healthcare professionals. This suggests that improved knowledge of labor and postpartum warning indicators will significantly improve BPCR practices.

### Strengths and Limitations of the Study

The inclusion of both community- and institutional-level studies is one of the strengths of this study. This review employs a systematic review and meta-analysis approach to estimate national predictors of BPCR practice in Ethiopia for the first time; healthcare policymakers, researchers, and practitioners may find important information from this work. Because there was insufficient research in certain regions, the results might not apply to the entire country. Only cross-sectional research was included in our analysis because no studies employing alternative methodologies were found. As a result, we suggest more studies to address these gaps.

### Conclusion

In this systematic review and meta-analysis, place of residence, maternal education, maternal knowledge of BPCR, antenatal care visit, history of stillbirth, knowledge of danger signs during pregnancy, knowledge of danger signs during labor and delivery, and knowledge of danger signs after delivery were the main identified factors affecting BPCR practices. Some of the variables were related to a lack of knowledge about maternal complications and were modifiable factors related to BPCR practices. Improving ANC services, improving women’s education, and integrating counseling into health facilities may lead to better BPCR practices. The findings of this study may help the policymaker design a policy to overcome the problems and increase overall maternal and neonatal health outcomes. Also, the findings of this review may be used as a baseline for future researchers who are interested in this field.
