# Supplementary material for: Predictors of Birth Preparedness and Complication Readiness Practices Among Pregnant Women in Ethiopia, a Systematic Review and Meta-Analysis
Source: Int J Public Health. 2024 Sep 2;69:1607296. doi: 10.3389/ijph.2024.1607296 (PMC11404039; doi:10.3389/ijph.2024.1607296)
Supplement: Supplementary file 2 [file DataSheet7.docx]

1. Funnel plot between Residency and practice of birth preparedness and complication readiness

1. Funnel plot between the educational status of women and practice of birth preparedness and complication readiness

1. Funnel plot between Knowledge of birth preparedness and complication readiness and practice of birth preparedness and complication readiness

1. Funnel plot between Knowledge of danger signs during pregnancy and practice of birth preparedness and complication readiness

1. Funnel plot between antenatal care follow-up and practice of birth preparedness and complication readiness

1. Funnel plot between History of Stillbirth and practice of birth preparedness and complication readiness

1. Funnel plot between Knowledge of Labor and delivery danger signs and practice of birth preparedness and complication readiness

1. Funnel plot between Knowledge of postpartum danger signs and practice of birth preparedness and complication readiness
