# Supplementary material for: Predictors of Birth Preparedness and Complication Readiness Practices Among Pregnant Women in Ethiopia, a Systematic Review and Meta-Analysis
Source: Int J Public Health. 2024 Sep 2;69:1607296. doi: 10.3389/ijph.2024.1607296 (PMC11404039; doi:10.3389/ijph.2024.1607296)
Supplement: Supplementary file 3 [file DataSheet6.docx]

1. Subgroup analysis based on study region for Knowledge on birth preparedness and complication readiness and birth preparedness and complication readiness practice

1. Subgroup analysis based on study design for Knowledge of birth preparedness and complication readiness and birth preparedness and complication readiness practice

1. Subgroup Analysis Based on the Study region for the history of stillbirth and birth preparedness and complication readiness practice

1. Subgroup Analysis Based on Study Design for the History of still Birth and birth preparedness and Complication Readiness Practice

1. Subgroup Analysis Based on Study region for Knowledge of danger signs during Pregnancy and birth preparedness and complication readiness practice

1. Subgroup Analysis Based on Study design for Knowledge of danger signs during Pregnancy and birth preparedness and complication readiness practice

1. Subgroup Analysis Based on Study region for Knowledge of danger signs during Labor and delivery and birth preparedness and complication readiness practice

1. Subgroup Analysis Based on Study design for Knowledge of danger signs during Labor and delivery and birth preparedness and complication readiness practice

1. Subgroup Analysis Based on Study region for Knowledge of postpartum danger signs and birth preparedness and complication readiness practice.

1. Subgroup Analysis Based on Study Design for Knowledge of postpartum danger signs and birth preparedness and complication readiness practice.
