# Supplementary material for: Predictors of Birth Preparedness and Complication Readiness Practices Among Pregnant Women in Ethiopia, a Systematic Review and Meta-Analysis
Source: Int J Public Health. 2024 Sep 2;69:1607296. doi: 10.3389/ijph.2024.1607296 (PMC11404039; doi:10.3389/ijph.2024.1607296)
Supplement: Supplementary file 6 [file DataSheet2.docx]

Supplementary File 2: The overall pooled odds ratio of the association between history of still birth and birth preparedness and complication readiness practice among pregnant Women in Ethiopia,2023.
